# A File Carrier

**Published:** 1866-06

**Authors:** 


### A FILE CARRIER.

Was invented, constructed and used by Dr. W. G. Redman, of Louisville Ky., for many years, without having made any effort to have it known to the profession even by a simple description, so far as we know. We feel that we have lost much on account of not having this instrument for the past ten years. It is certainly altogether the best file carrier we have ever seen. It can be reversed so as to serve the purpose of two ordinary carriers. Is especially adapted for use upon the molar teeth, may be used upon the front as well. We will not attempt a description of the instrument but will simply say that it has got out, and may be obtained at the Dental Depots, and every dentist ought to have one.

